# Supplementary material for: Effects of Polymer Blending on the Performance of a Subcutaneous Biodegradable Implant for HIV Pre-Exposure Prophylaxis (PrEP)
Source: Int J Mol Sci. 2021 Jun 18;22(12):6529. doi: 10.3390/ijms22126529 (PMC8235439; doi:10.3390/ijms22126529)
Supplement: Supplementary file 1 [file ijms-22-06529-s001.zip › ijms-1228267-supplementary.pdf]

Supplementary Materials: Effects of Polymer Blending on the Performance of a Subcutaneous Biodegradable Implant for HIV Pre-Exposure Prophylaxis (PrEP)

Linying Li [1], Christine Areson [1], Ariane van der Straten [2,3], and Leah M. Johnson [1 *]

1 Engineered Systems RTI International, 3040 E. Cornwallis Road, Durham, North Carolina 27709, USA; ali@rti.org (L.L.); careson@rti.org (C.A.)

2 Center for AIDS Prevention Studies, Dept Department of Medicine, University of California San Francisco, San Francisco, California, 94104, USA; arianevds@gmail.com

3 ASTRA consulting, Kensington, California CA 94708, USA

* Correspondence: leahjohnson@rti.org; Tel.: +1-919-541-7233

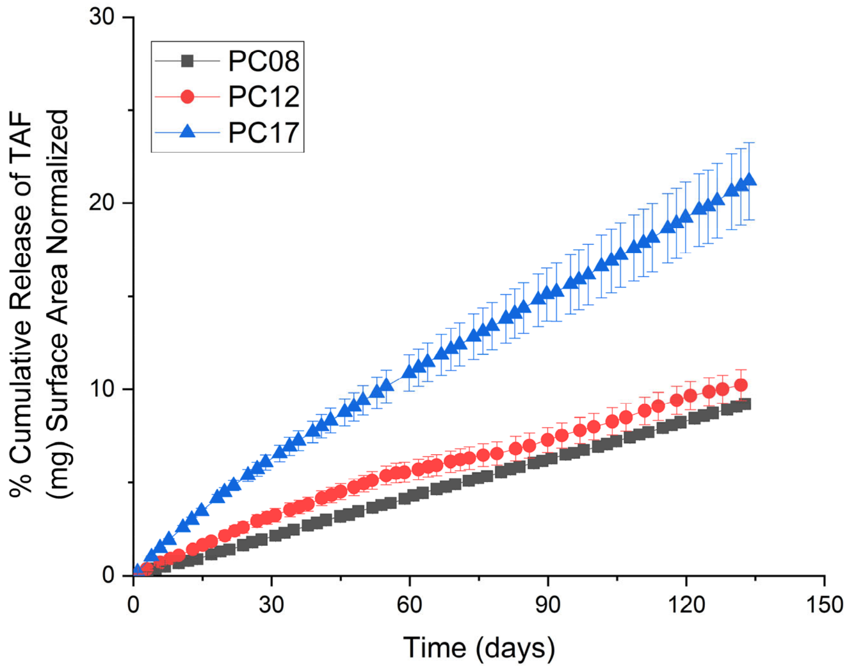

Figure S1. Percentage Cumulative release profiles of TAF from implants comprising extruded tubes of PC-08, PC-12, and PC-17. All implants contain a formulation of 2:1 TAF/ sesame oil and tubes with a wall thickness of 100 μm, a length of 40 mm, and an OD of 2.5 mm. All samples were performed in triplicate.

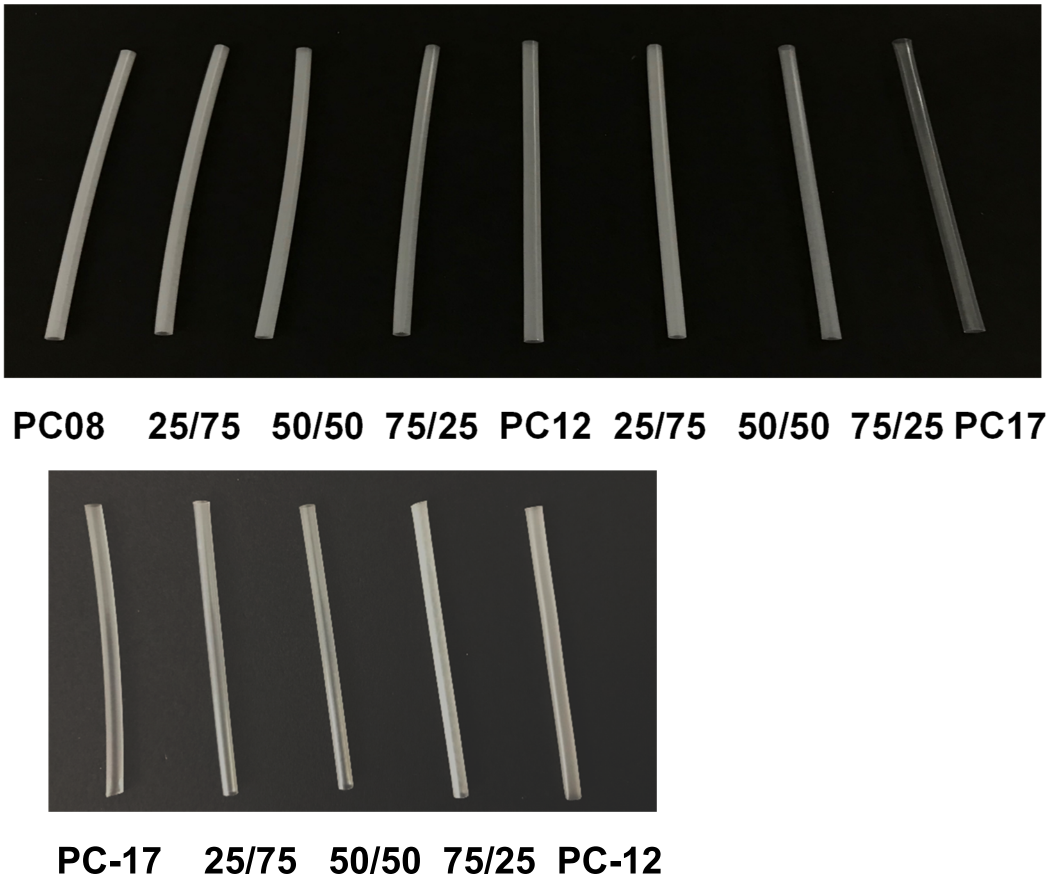

Figure S2. Digital camera image of extruded tubes comprising pure PCL formulations and blends.

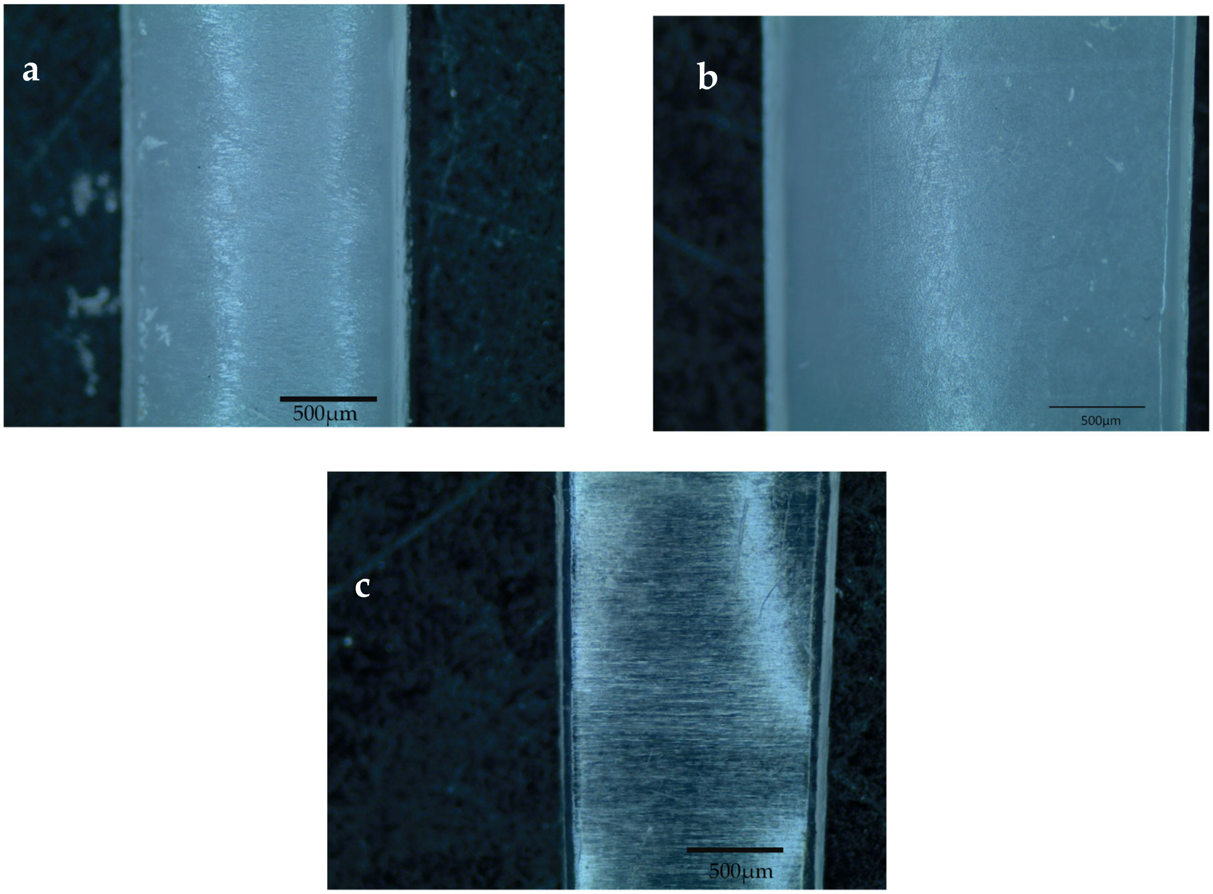

Figure S3. The microscope images of the varying 100 μm PCL tubes: (a) PC-08 100 μm, (b) PC-12 100 μm, and (c) PC-17 100 μm. The extruded tubes were sliced open and flattened to adhere to glass slides. The magnification of the microscope is set at 4.5 times.

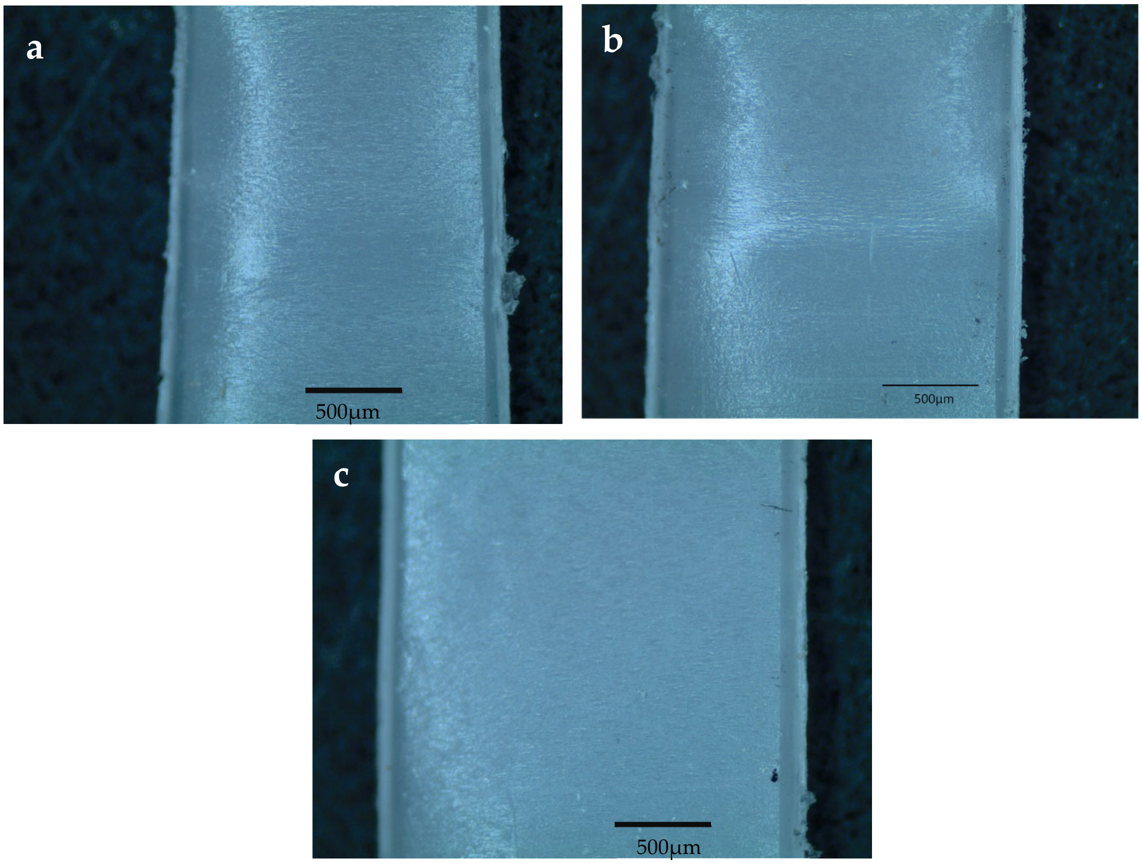

Figure S4. The microscope images of the binary PC-08/PC-12 blends: (a) PC-08/ PC-12 25/75, (b) PC-08/ PC-12 50/50, and (c) PC-08/ PC-12 75/25. The wall thickness of the extruded tubes comprising these blends is 100 μm. The extruded tubes were sliced open and flattened to adhere to glass slides. The magnification of the microscope is set at 4.5 times.

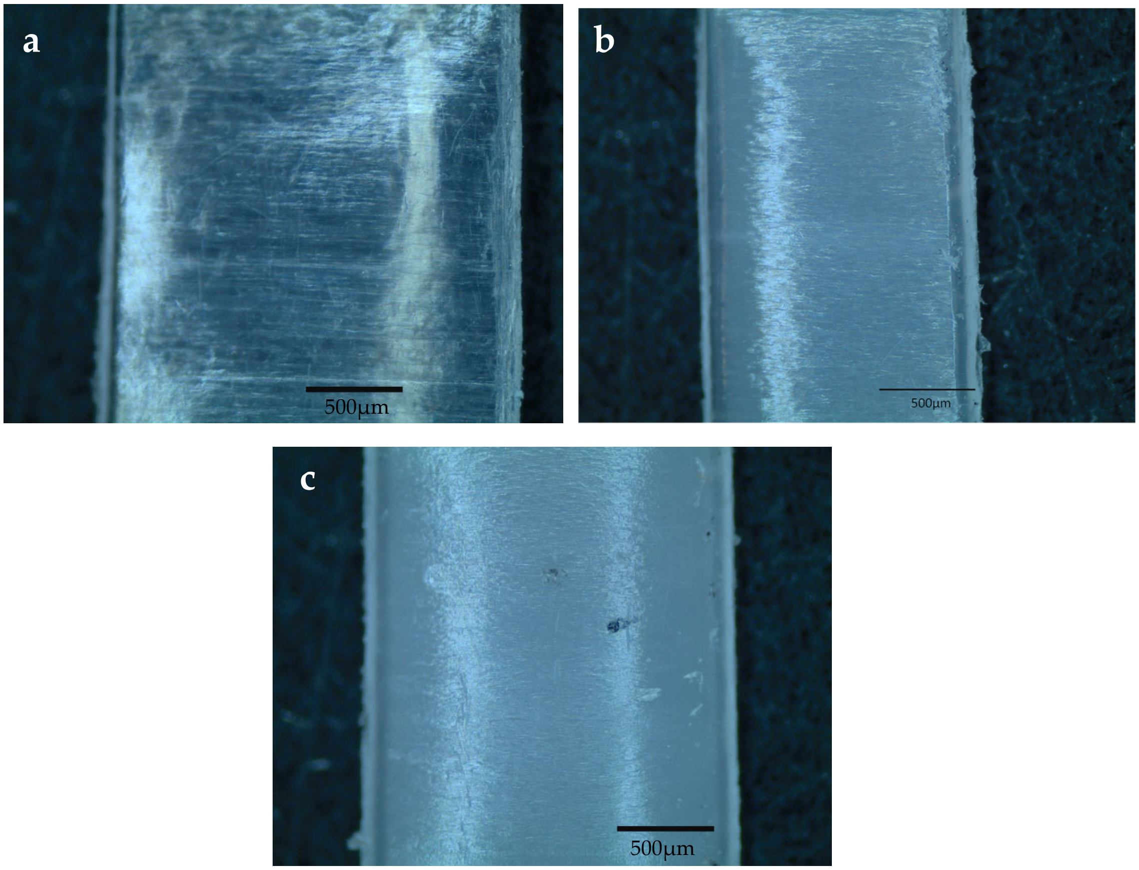

Figure S5. The microscope images of the binary PC-08/PC-17 blends: (a) PC-08/ PC-17 25/75, (b) PC-08/ PC-17 50/50, and (c) PC-08/ PC-17 75/25. The wall thickness of the extruded tubes comprising these blends is 100 µm. The extruded tubes were sliced open and flattened to adhere to glass slides. The magnification of the microscope is set at 4.5 times.

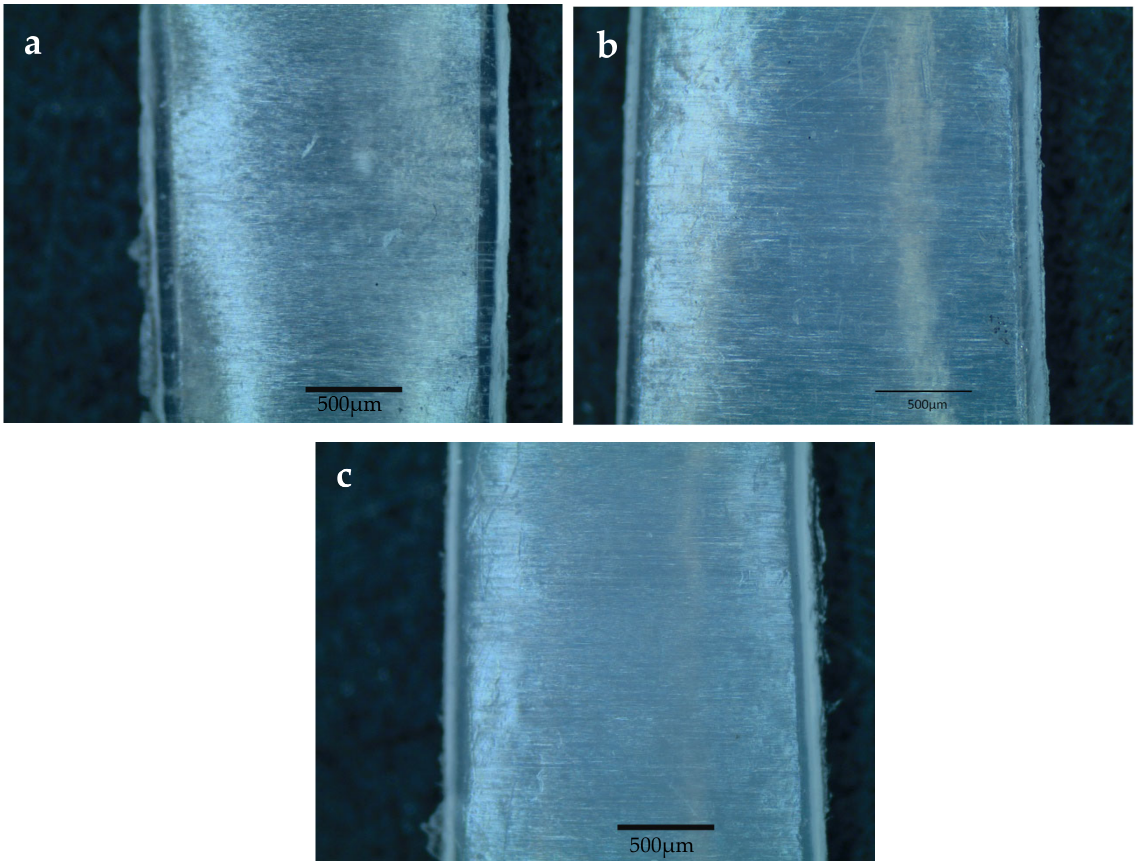

Figure S6. The microscope images of the binary PC-12/PC-17 blends: (a) PC-12/ PC-17 25/75, (b) PC-12/ PC-17 50/50, and (c) PC-12/ PC-17 75/25. The wall thickness of the extruded tubes comprising these blends is 100 μm. The extruded tubes were sliced open and flattened to adhere to glass slides. The magnification of the microscope is set at 4.5 times.

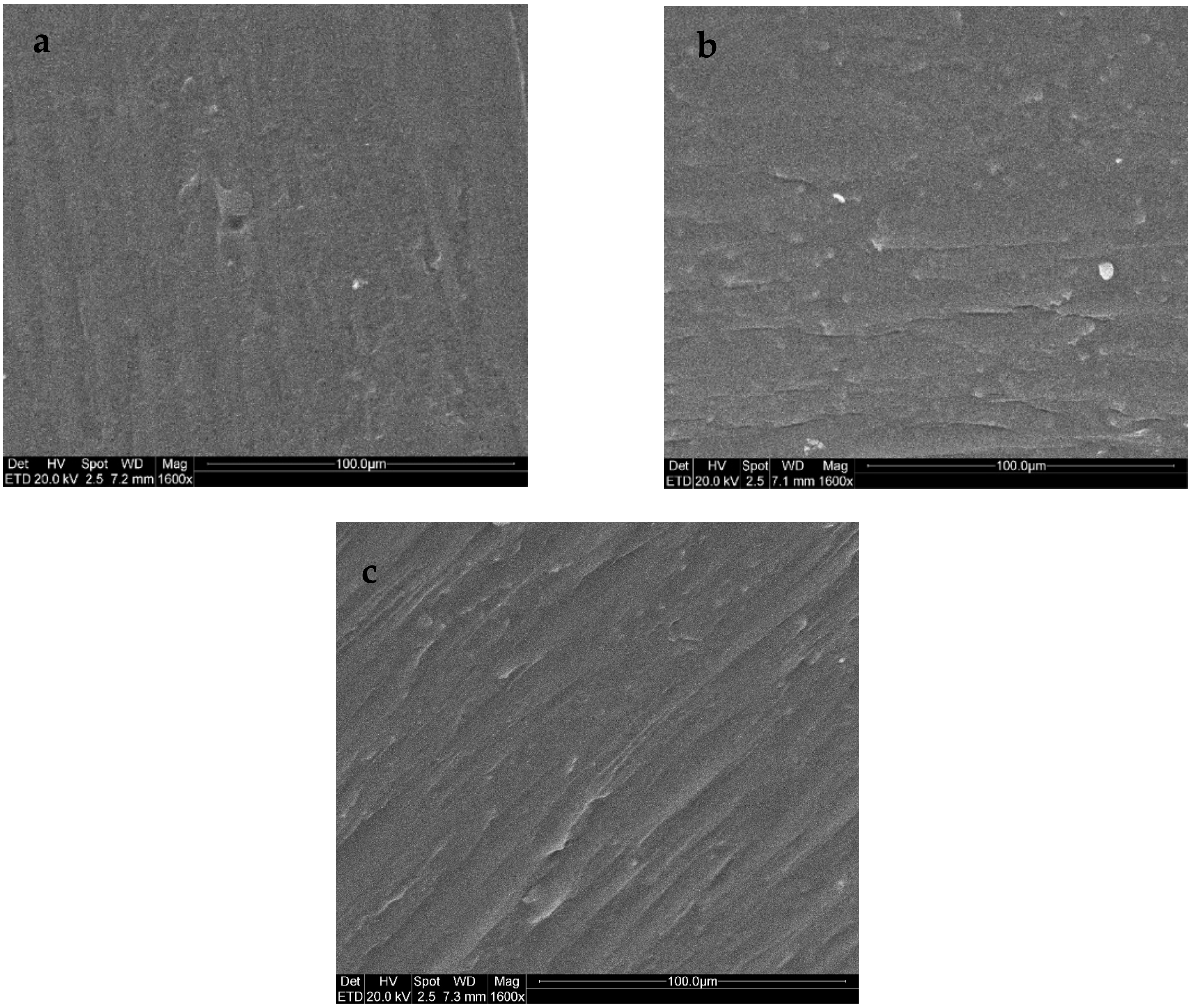

Figure S7. The SEM images of the binary PC-08/PC-12 blends: (a) PC-08/ PC-12 25/75, (b) PC-08/ PC-12 50/50, and (c) PC-08/ PC-12 75/25. The wall thickness of the extruded tubes comprising these blends is 100 μm. Samples were prepared for imaging by slicing the extruded tubes and flattened them onto a carbon adhesive substrate. The SEM images were taken at a voltage of 20 kV and a spot size of 2.5 under high vacuum at magniciation of 1600K.

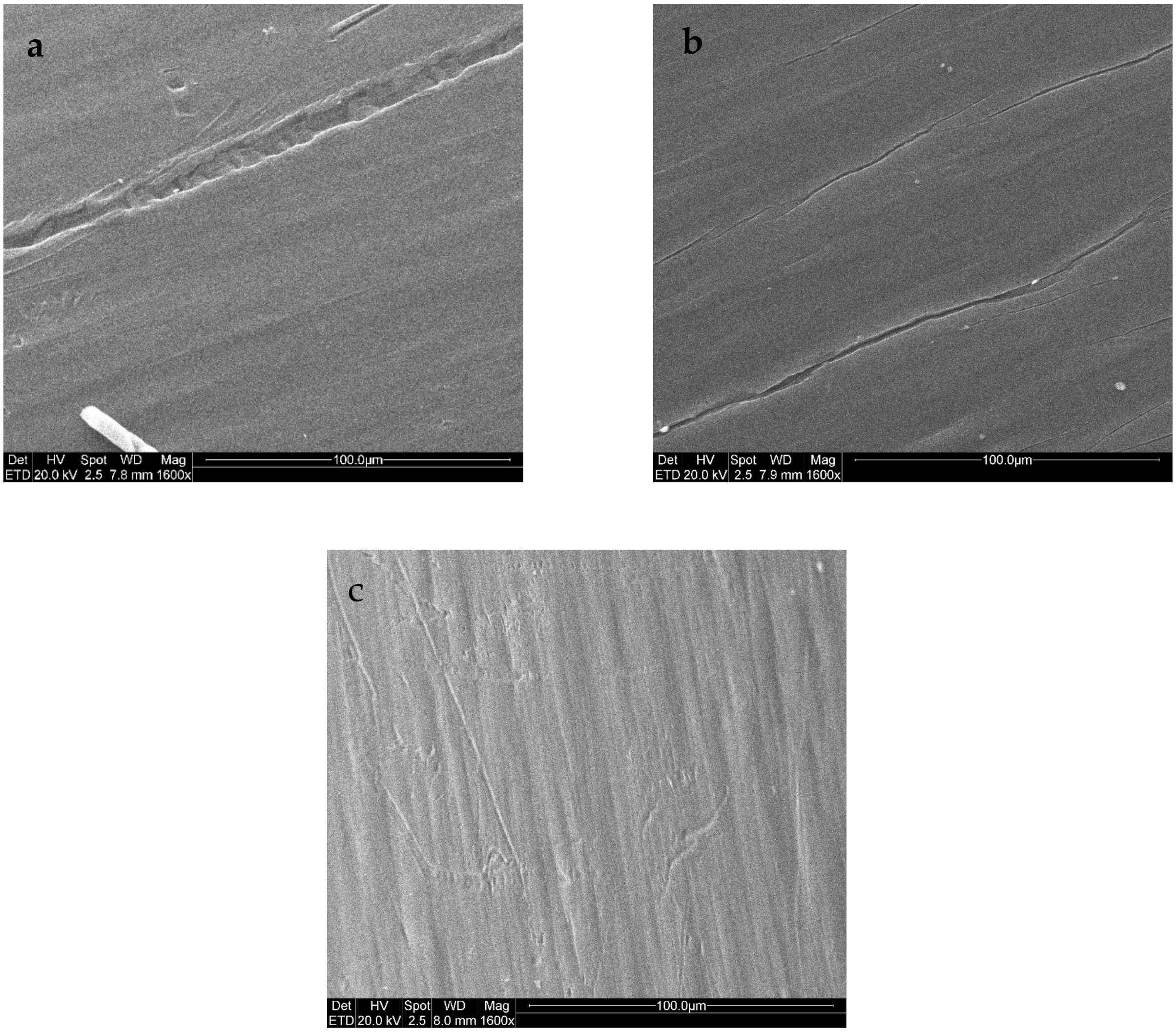

Figure S8. The SEM images of the binary PC-08/PC-17 blends: (a) PC-08/ PC-17 25/75, (b) PC-08/ PC-17 50/50, and (c) PC-08/ PC-17 75/25. The wall thickness of the extruded tubes comprising these blends is 100 μm. Samples were prepared for imaging by slicing the extruded tubes and flattened them onto a carbon adhesive substrate. The SEM images were taken at a voltage of 20 kV and a spot size of 2.5 under high vacuum at magniciation of 1600K.

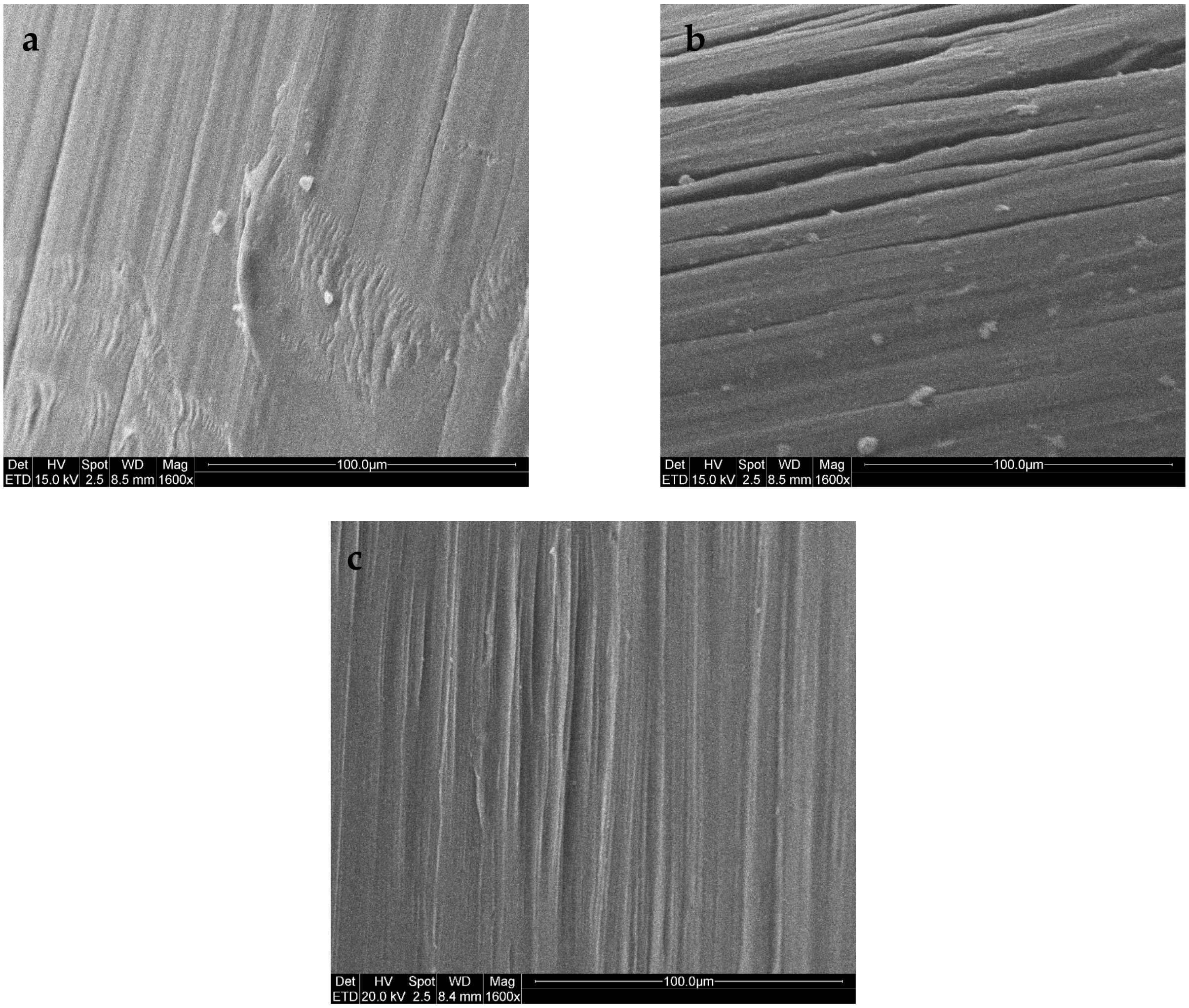

Figure S9. The SEM images of the binary PC-12/PC-17 blends: (a) PC-12/ PC-17 25/75, (b) PC-12/ PC-17 50/50, and (c) PC-12/ PC-17 75/25. The wall thickness of the extruded tubes comprising these blends is 100 μm. Samples were prepared for imaging by slicing the extruded tubes and flattened them onto a carbon adhesive substrate. The SEM images were taken at a voltage of 20 kV and a spot size of 2.5 under high vacuum at magniciation of 1600K.

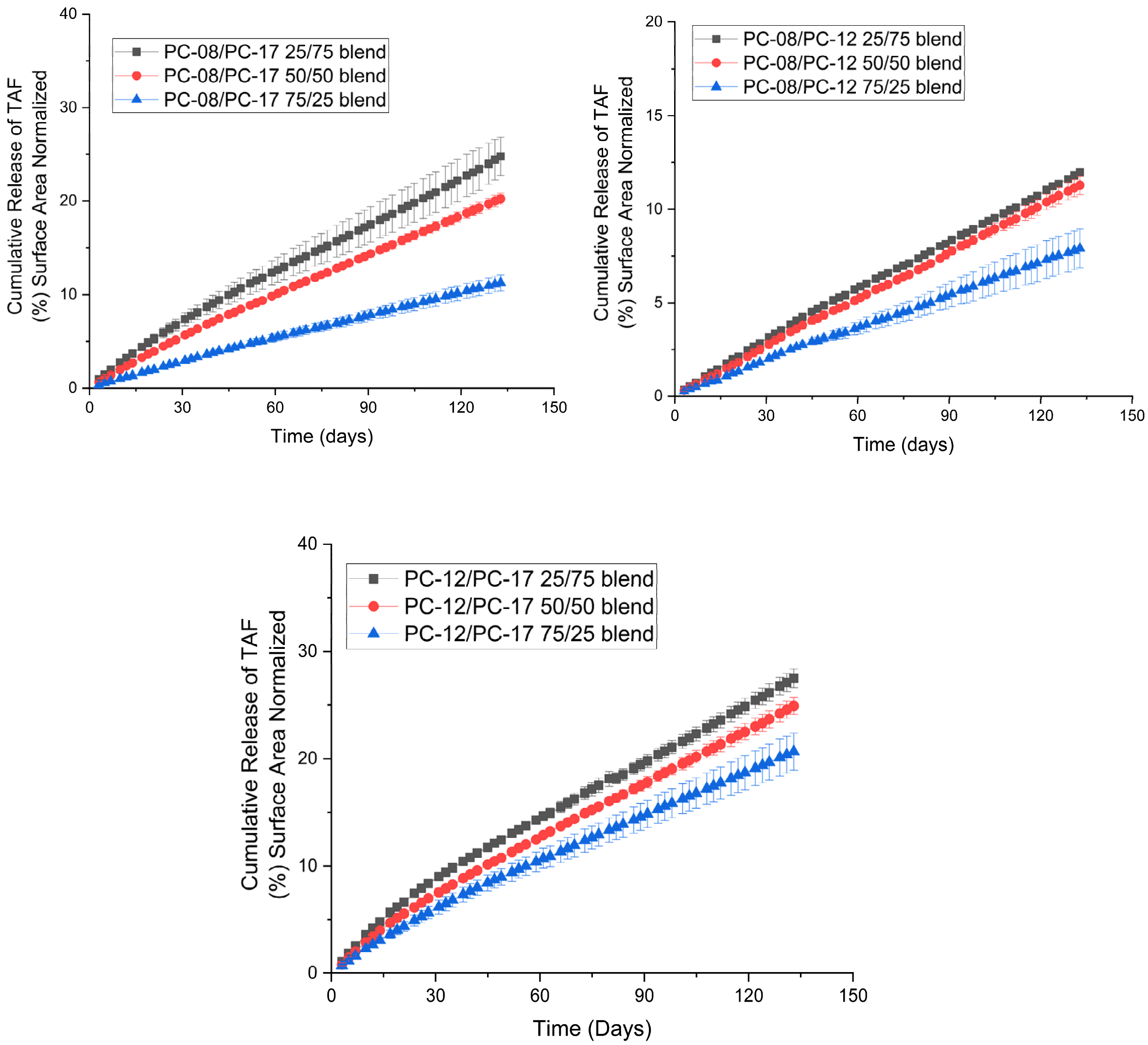

Figure S10. Percentage Cumulative release profiles of TAF from implants comprising extruded tubes of PCL MW blends. All implants contain a formulation of 2:1 TAF/ sesame oil and tubes with a wall thickness of 100 µm, a length of 40 mm, and an OD of 2.5 mm. All samples were performed in triplicate.
